# An OsGLK1/2-DGP1/2/3 feedback module regulates rice photosynthesis and yield

**DOI:** 10.1126/sciadv.aef5234

**Published:** 2026-06-24

**Authors:** Xueju Liu, Kangwei Liu, Shu Xu, Jiaqi Tang, Jinglin Huang, Li Deng, Chao Zhang, Hengxiu Yu

**Affiliations:** ^1^ Jiangsu Key Laboratory of Crop Genomics and Molecular Breeding/Zhongshan Biological Breeding Laboratory/Key Laboratory of Plant Functional Genomics of the Ministry of Education/Jiangsu Co-Innovation Center for Modern Production Technology of Grain Crops, Agricultural College of Yangzhou University, Yangzhou, China.; ^2^ Yangzhou Modern Seed Innovation Institute, Gaoyou, China.

## Abstract

GOLDEN2-LIKE (GLK) proteins are central regulators of plant photosynthesis, and their molecular mechanisms are not fully elucidated, especially in rice. Here, we report a GLK-centered molecular module that regulates rice photosynthesis and yield. *Osglk1 Osglk2* double mutants are seedling lethal, and *Osglk2* presents faded leaves and white panicles, which indicated the partially redundant role of *OsGLK1* and *OsGLK2* in photosynthesis regulation. Transcriptome sequencing analysis indicated that OsGLK regulates the expression of genes related to carbohydrate metabolism and photosynthesis. Overexpression of *OsGLK1*/*2* results in dark-green–panicle phenotypes. We proved that DEEP GREEN PANICLE2 (DGP2) and DGP3, homologs of DGP1, are direct repressors of OsGLK activity and function negatively in rice chlorophyll content regulation. Moreover, OsGLK directly binds to the promoters of *DGP1*/*2*/*3* and activates their expressions. Agronomic trait analysis showed that mutations of *DGP1* and *DGP2* significantly increased seed weight and yield of rice. Together, these results manifested an OsGLK1/2-DGP1/2/3 feedback module regulating rice photosynthesis and yield and may provide approaches for crop breeding.

## Teaser

OsGLK1/2 activate the expressions of DGP1/2/3, and DGP1/2/3 suppress the activities of OsGLK1/2 to orchestrate photosynthesis.

## INTRODUCTION

Photosynthesis converts light energy into chemical energy and lays the foundation for all life activities on the earth. Photosynthesis occurs within chloroplasts, a semiautonomous organelle that contains DNA and gene expression apparatuses (*1*). Thus, the coordination of photosynthesis necessitates proper expressions of photosynthesis-associated nuclear genes (PhANGs) and plastid genes. The regulations of PhANGs are largely controlled by light and hormonal signals, in which transcription factors, such as GOLDEN2-LIKE (GLK), GATA NITRATE-INDUCIBLE CARBON METABOLISM–INVOLVED, and CYTOKININ-RESPONSIVE GATA FACTOR 1, exerted functions (*2*–*4*).

GLK proteins exist as pairs (GLK1 and GLK2) in most characterized plant genomes and are GOLDEN2, Arabidopsis RESPONSE REGULATOR-B (ARR), and PHOSPHATE STARVATION RESPONSE1 (Psr1) transcription factors that play pleiotropic functions in diverse plant species (*5*, *6*). The predominant function of GLK protein is involved in regulating genes related to photosynthesis and chloroplast biogenesis. Mutant of *Golden 2*, the *GLK2* homolog in maize, displays pale green leaf blades and white leaf sheaths (*7*). Double mutants of *GLK1* and *GLK2* are pale green with perturbed chloroplast development in *Arabidopsis thaliana* and *Physcomitrella patens*. Expression level of genes related to light harvesting and chlorophyll biosynthesis is down-regulated (*8*, *9*). Constitutive *GLK* gene expression leads to increased accumulation of transcripts for antenna proteins and chlorophyll biosynthetic enzymes by direct promoter binding (*10*, *11*). Similarly, *Osglk1 Osglk2* of rice, generated by introducing an RNA interference (RNAi) construct of *OsGLK1* into transferred DNA (T-DNA) insertion line of *OsGLK2*, is phenotypically pale, and the overexpression of *OsGLK1* induces chloroplast development in nongreen rice cells (*12*–*14*). Mutation of *GLK* in *Cucumis melo*, in which only one copy of *GLK* was identified, leads to yellow-green plant and reduced chloroplast number (*15*). Functional characterization of GLKs in chloroplast developments and chlorophyll synthesis was also reported in other plants, including tomato, peach, lettuce, and barley (*16*–*19*). On the basis of the conserved functions in photosynthesis regulation, GLKs have been implicated in boosting crop yields (*20*–*22*). Moreover, GLKs also participate in other developmental processes of plants, such as fruit nutrition, fertility, and responses to stress (*23*–*29*).

The activities of GLKs are regulated in multiple levels, including transcription of *GLKs*, protein interaction, and protein degradation (*14*, *30*–*32*). In particular, a plant-specific protein family containing TIGR01589 domain is emerging as regulators of GLK activity. Although differentially named as *PSEUDO-ETIOLATION IN LIGHT* (*PEL*), *REPRESSOR OF PHOTOSYNTHETIC GENES* (*RPGE*), and *Brz-INSENSITIVE-PALE GREEN* (*BPG*) in *Arabidopsis* by different researchers, they are actually the same genes (*33*–*36*). Plants with overexpression of *PEL1* (also named as *RPGE2* and *BPG4*) showed the pale green phenotype and fast plant development and grew taller than wild-type plants, which is reminiscent of wild-type plants grown under weak light conditions (*33*). Similarly, overexpression of *TkPEL-like* in *Taraxacum koksaghyz* causes a pale green phenotype (*37*). Overexpression of *PEL2* (also names as *RPGE1* and *BGH2*) also leads to pale green leaves (*34*). Both PEL1 and PEL2 interact with GLK1/2 and inhibit their DNA binding activities (*38*). Editing of rice *PEL* microProtein genes promotes chloroplast development (*39*). Insertional mutagenesis of a *PEL* homolog from carrot (*Daucus carota*) appears to induce the accumulation of carotenoids in taproots (*40*). This induction might also dependent on the activity release of GLKs, which are proved to directly regulate carotenoid biosynthesis in *Arabidopsis* (*29*).

We previously characterized DGP1 (DEEP GREEN PANICLE1), a rice homolog of PEL1, which suppresses GLK activity to reduce chlorophyll synthesis in rice glumes (*41*). In this study, we found an OsGLK1/2-DGP1/2/3 feedback module, in which OsGLK1/2 directly activate the expression of *DGP1*/*2*/*3* and DGP1/2/3 directly repress the activities of OsGLK1/2, which regulates rice photosynthesis and yield.

## RESULTS

### *OsGLKs* regulate chlorophyll synthesis and chloroplast development in a partially redundant manner

To examine the functions of *OsGLK1/2* in photosynthetic pigment biosynthesis and chloroplast development, we first created their mutants using gene editing approach and revisited the phenotypes. After genetic transformation, propagating, and genotyping, we obtained single mutants (*Osglk1-1* and *Osglk1-2* for *OsGLK1*, *Osglk2-1* and *Osglk2-2* for *OsGLK2*) and corresponding double mutants (*Osglk1-1 Osglk2-1* and *Osglk1-2 Osglk2-2*) (fig. S1). Different alleles for single and double mutants present same phenotype, and *Osglk1-1*, *Osglk2-1*, and *Osglk1-1 Osglk2-1* were used for following studies.

**Fig. 1.**
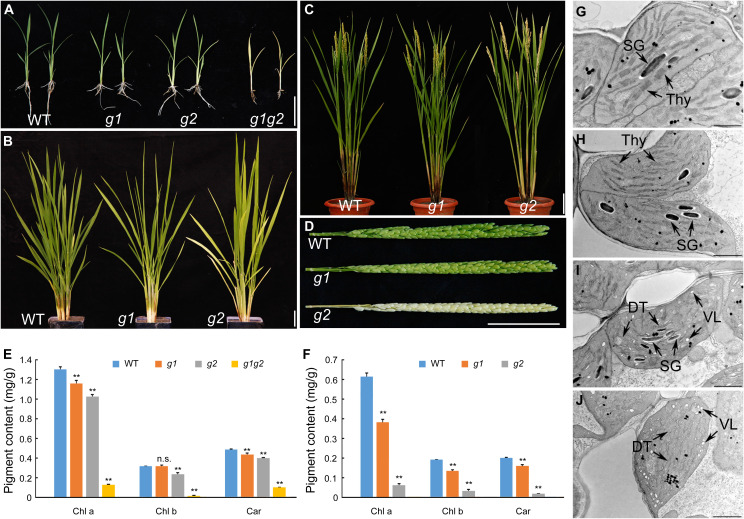
Phenotypic characterization of *OsGLK* mutants. (**A**) Phenotypes of wild type (WT), *Osglk1* (*g1*), *Osglk2* (*g2*), and *Osglk1 Osglk2* (*g1g2*) at seedling stage. Scale bar, 5 cm. (**B**) Phenotypes of WT, *g1*, and *g2* at tillering stage. Scale bar, 10 cm. (**C**) Phenotypes of WT, *g1*, and *g2* at mature stage. Scale bar, 10 cm. (**D**) Panicles of WT, *g1*, and *g2*. Scale bar, 5 cm. (**E** and **F**) Photosynthetic pigment content of seedlings (E) and panicles (F). Data are presented as the mean ± SD. Asterisks indicate significant differences according to a two-tailed Student’s *t* test (**P < 0.01). n.s., not significant. (**G** to **J**) Transmission electron microscopy (TEM) images of the leaves of WT (G), *Osglk1* (H), *Osglk2* (I), and *Osglk1 Osglk2* (J). DT, disintegrated thylakoid; SG, starch granule; Thy, thylakoid; VL, vesicle-like structure. Scale bars, 10 μm.

At seedling stage, *Osglk1 Osglk2* is yellow, which is consistent with previous study (Fig. 1A) (*13*). Unexpectedly, we found that the seedlings of *Osglk2* are light yellow, whereas *Osglk1* is indistinguishable from wild type. Accordingly, photosynthetic pigment contents of *Osglk1 Osglk2* and *Osglk2* are significantly less than that of wild type and only slightly reduced in *Osglk1* (Fig. 1E). *Osglk1 Osglk2* gradually wilts and dies within 4 weeks, which may due to deficient photosynthesis. With growth and development, *Osglk2* consistently presents light-yellow leaves (Fig. 1B). At the heading stage, *Osglk2* presents white panicles, and the color of *Osglk1* panicles slightly faded, which is consistent with the photosynthetic pigment contents (Fig. 1, C, D, and F).

Ultrastructure of chloroplasts in leaves from wild-type and mutants was investigated by transmission electron microscopy (TEM). In the wild type, fully developed chloroplasts with massive thylakoid membranes and starch granules were observed (Fig. 1G). Chloroplast structure of *Osglk1* is similar with that of wild type (Fig. 1H). In *Osglk2* chloroplast, thylakoid membrane seems less organized with swollen lamellae and vesicle-like structures (Fig. 1I). In *Osglk1 Osglk2* chloroplast, thylakoid membranes are completely disintegrated to monolayer swollen lamellae. Moreover, starch granules were never observed (Fig. 1J). These above results indicate that *OsGLK1* and *OsGLK2* regulate chlorophyll synthesis and chloroplast development in a partially redundant manner.

### OsGLK regulates the expression of genes related to carbohydrate metabolism and photosynthesis

**Fig. 2.**
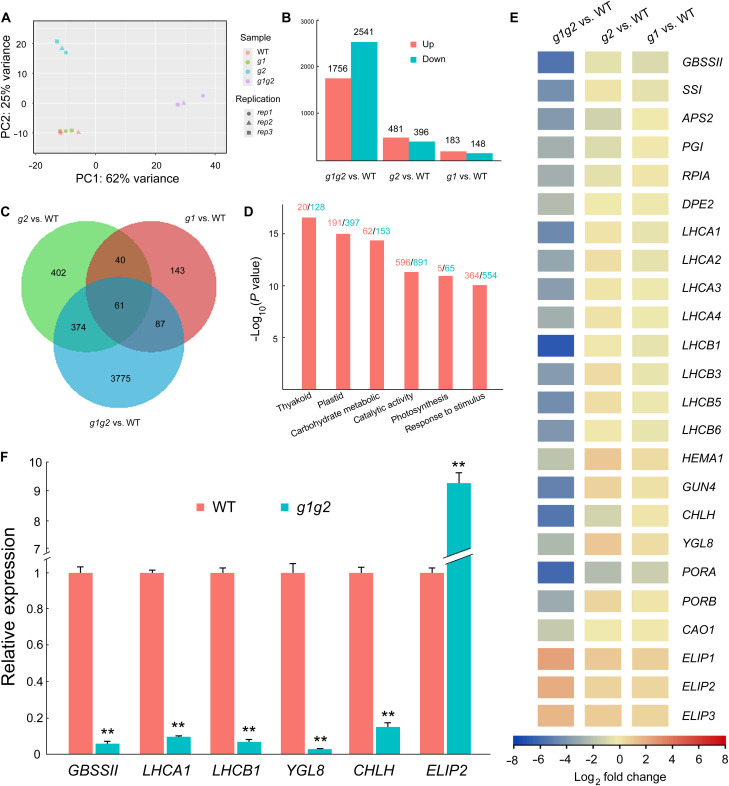
Expression analysis of genes regulated by *OsGLK*. (**A**) PCA of transcriptome sequencing data from WT, *g1*, *g2*, and *g1g2*. Colors and shapes represent samples and replications, respectively. (**B**) Statistical analysis of DEGs between mutants and WT. The number of genes in each comparison is indicated. (**C**) Venn diagram of shared and distinct DEGs in each comparison. (**D**) Top six enriched Gene Ontology (GO) items in DEGs between WT and *g1g2*. The number of up- and down-regulated genes in each item is indicated. (**E**) Heatmap of the fold changes (log_2_) in the expression of carbohydrate metabolism and photosynthesis-related genes in each comparison. Data of normalized read counts from transcriptome analysis were used to calculate the expression levels. (**F**) Validation of RNA sequencing (RNA-seq) data using quantitative reverse transcription polymerase chain reaction (qRT-PCR). Data are presented as the mean ± SD. Asterisks indicate significant differences according to a two-tailed Student’s *t* test (**P < 0.01).

To further dissect the biological function of OsGLK in rice development, we carried out a transcriptome analysis between wild type and mutants, including *Osglk1*, *Osglk2*, and *Osglk1 Osglk2*. Principal components analysis (PCA) of transcriptome sequencing data showed that replications of *Osglk2* and *Osglk1 Osglk2* gathered respectively while wild type and *Osglk1* mixed together (Fig. 2A). Compared with wild type, 1756 genes are up-regulated and 2541 genes are down-regulated in *Osglk1 Osglk2*. The number of up- and down-regulated genes decreased to 481 and 396 in *Osglk2* and 183 and 148 in *Osglk1* (Fig. 2B). Both the PCA and number of differentially expressed genes (DEGs) are in accordance with the severities of the mutant phenotypes (Fig. 1). Nearly half of the DEGs identified in *Osglk1* (44.71%) and *Osglk2* (49.60%) are overlapped with the ones identified in *Osglk1 Osglk2* (Fig. 2C).

As revealed by the Gene Ontology (GO) analysis, genes involved in carbohydrate metabolic process, photosynthesis, and response to stimulus are markedly enriched in DEGs of *Osglk1 Osglk2* (Fig. 2D). Notably, among the 70 DEGs in photosynthesis, 65 genes are down-regulated, and only 5 genes are up-regulated. Detailed analysis of some DEGs in carbohydrate metabolic process and photosynthesis showed that they are regulated by OsGLK1 and OsGLK2 in a redundant or partially redundant way (Fig. 2, E and F). For example, the expression level of *OsPORA*, a gene involved in chlorophyll synthesis, in *Osglk1 Osglk2* is 1.51% of the wild type. This ratio in *Osglk2* and *Osglk1* is 21.58% and 37.00, respectively. Three early light-induced protein (ELIP) genes, which were identified as genes rapidly transcribed after etiolated seedlings were transferred from the dark to the light, are up-regulated in mutants. The genes (*PAP1*-*PAP13*) encoding plastid-encoded RNA polymerase–associated proteins (PAPs) are up-regulated in *Osglk1 Osglk2*, which may reflect a feedback regulation of photosynthesis-related gene expression (fig. S2).

### Overexpression of *OsGLK1/2* results in a dark-green panicle phenotype

**Fig. 3.**
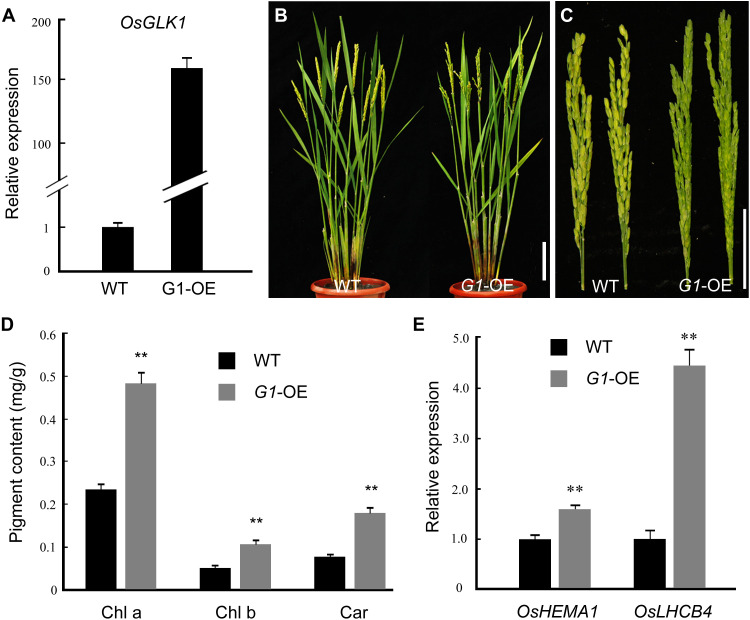
Overexpression of *OsGLK1* results in a dark-green–panicle phenotype. (**A**) Relative expression levels of *OsGLK1* in WT and *G1*-OE (*OsGLK1* overexpression). (**B**) Comparison of the plants of WT and *G1*-OE. Scale bar, 10 cm. (**C**) Comparison of the panicles of WT and *G1*-OE. Scale bar, 5 cm. (**D**) Chlorophyll content in glumes of WT and *G1*-OE. Mean and SD values were obtained from three biological replicates. (**E**) Expression analysis of *OsHEMA1* and *OsLHCB4* in WT and *G1*-OE glumes. Asterisks indicate significant differences according to a Student’s *t* test (**P < 0.01).

To confirm the regulation role of *OsGLK* in rice photosynthesis, we generated *OsGLK1*-overexpressing lines (*G1*-OE) driven by the rice actin promoter. Quantitative reverse transcription polymerase chain reaction (qRT-PCR) assay verified the accumulation of *OsGLK1* transcripts in *G1*-OE (Fig. 3A). Plants of *G1*-OE showed comparable morphology and leaf color with wild type (Fig. 3B). However, the panicles of *G1*-OE are greener than those of the wild type (Fig. 3C). Accordingly, the glumes of *G1*-OE had significantly more photosynthetic pigment than that of the wild type (Fig. 3D). Chlorophyll synthesis–related genes *HEMA1* and antenna protein gene *LHCB4* are target genes of *GLK*, and their expressions are significantly up-regulated in *G1*-OE (Fig. 3E). We also obtained the *OsGLK2*-overexpressing lines, and a same dark-green–panicle phenotype was observed (fig. S3).

### DGP2/3 are direct repressors of OsGLK activity

**Fig. 4.**
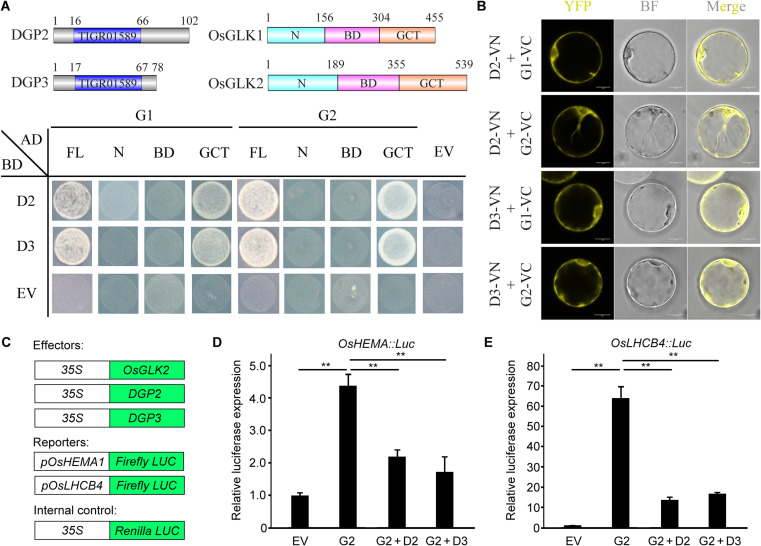
DGP2/3 directly repress the transcriptional activity of OsGLK. (**A**) DGP2/3 interact with GLK/C-terminal (GCT) domain of OsGLK1 and OsGLK2 in yeast two-hybrid assays. The indicated combinations of constructs were cotransformed into yeast cells and grown on the selective quadruple dropout medium. Schematic diagrams of the protein structures of DGP2/3 and OsGLK are shown. AD, activation domain; BD, DNA binding domain; EV, empty vector; FL, full length. (**B**) DGP2/3 interact with OsGLK1 and OsGLK2 in bimolecular fluorescence complementation (BiFC) assay. BF, bright field; D2, DGP2; D3, DGP3. (**C**) Schematic diagram of construct used in transactivation activity assays. Promoters are shown as white boxes, and coding sequences (CDSs) are shown as green boxes. (**D** and **E**) Analysis of the effect of DGP2/3 on the transcription activity of OsGLK2 on promoter of *OsHEMA1* (D) and *OsLHCB4* (E). Asterisks indicate significant differences according to a Student’s *t* test (**P < 0.01).

In our previous study, we characterized rice DGP1 as a repressor of OsGLK activity and thus chlorophyll synthesis in rice (*41*). By searching rice genome, six genes with homology to *DGP1* were identified and named *DGP2* to *DGP7* individually (table S1). In particular, these genes could be divided into two groups based on the length of encoded proteins. DGP2 and DGP3, the first group, contained 102 and 78 amino acids, respectively, which are comparable with 102 amino acids of DGP1 (Fig. 4A). The protein length of the second group (DGP4, DGP5, DGP6, and DGP7) is relatively long, which ranges from 289 to 350 amino acids. However, the plant-specific domain, TIGR01589, is conserved across DGP1 to DGP7 (figs. S4 and S5).

Considering protein sequence similarity and protein length, we choose *DGP2* and *DGP3* for further study. Subcellular localization results show that DGP2 and DGP3 displayed a ubiquitous distribution pattern throughout the cell (fig. S6). We questioned whether DGP2/3, such as DGP1, interact with OsGLK. Yeast two-hybrid assay proved that both DGP2 and DGP3 interact with OsGLK1/2 (Fig. 4A). Furthermore, to determine the functional domains required for the interaction, we generated three truncated versions of OsGLK, which contained the N-terminal, DNA-BD (DNA binding domain), or GCT (GLK/C-terminal) box domain, as previously reported (*42*). Our results showed that DGP2/3 interact only with the GCT box domain of OsGLK1/2 (Fig. 4A). A bimolecular fluorescence complementation (BiFC) assay further shows the interactions between DGP2/3 and OsGLK1/2 both in the nucleus and the cytosol (Fig. 4B and fig. S7).

To investigate whether DGP2/3, such as DGP1, could inhibit OsGLK transcription factor activity, we performed luciferase-based transactivation assays in rice protoplasts using the promoters of the OsGLK target genes *OsHEMA1* (chlorophyll biosynthetic gene) and *OsLHCB4* (antenna protein gene) as reporter (Fig. 4C). As expected, OsGLK2 significantly activated the promoters of *OsHEMA1* and *OsLHCB4* (Fig. 4, D and E). However, this transcriptional activation was significantly decreased when the *35S::DGP2/3* effector was cotransfected with *35S::OsGLK2* (Fig. 4, D and E). These results indicate that DGP2/3 interact with OsGLK1/2 and repress their transcriptional activity. Meanwhile, the expression levels of *OsGLK1/2* in *dgp* mutants were unaffected (fig. S8).

### *DGP2* and *DGP3* function negatively in rice chlorophyll content regulation

**Fig. 5.**
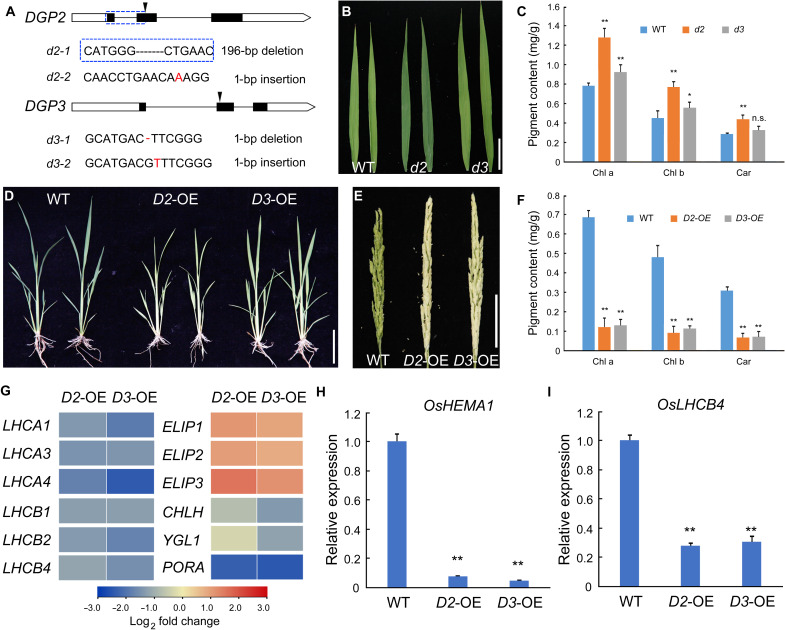
*DGP2/3* function in rice chlorophyll content regulation. (**A**) Genotypes of *dgp2* (*d2*) and *dgp3* (*d3*) mutant. The arrow head indicates the position of target sequence for CRISPR-Cas9. Details of sequence modification in each mutant are listed below the gene structure. (**B**) Comparison of the flag leaves from WT, *d2*, and *d3*. Scale bar, 5 cm. (**C**) Chlorophyll content in flag leaves of WT, *d2*, and *d3*. Mean and SD values were obtained from three biological replicates. (**D** and **E**) Comparison of the seedlings (D) and panicles (E) of WT, *D2*-OE, and *D3*-OE. Scale bars, 5 cm. (**F**) Chlorophyll content in glumes of WT, *D2*-OE, and *D3*-OE. Mean and SD values were obtained from three biological replicates. Asterisks indicate significant differences according to a Student’s *t* test (**P < 0.01). (**G**) Heatmap of the fold changes (log_2_) in the expression of photosynthesis-related genes in each comparison. Data of normalized read counts from transcriptome analysis were used to calculate the expression levels. (**H** and **I**) qRT-PCR analysis of the expression level of *OsHEMA1* (H) and *OsLHCB4* (I) in *D2*-OE and *D3*-OE. Data are presented as the mean ± SD. Asterisks indicate significant differences according to a two-tailed Student’s *t* test (**P < 0.01).

To analyze the physiological functions of *DGP2* and *DGP3*, we created corresponding mutants using gene editing approach. Two mutants for both *dgp2* (*d2-1* and *d2-2*) and *dgp3* (*d3-1* and *d3-2*) were obtained (Fig. 5A). In *d2-1*, a 196–base pair (bp) deletion, which contained the initiation codon, occurred. *d2-2* harbored a 1-bp insertion, which putatively led to shift of open reading frame. For *dgp3*, 1-bp deletion in *d3-1* and 1-bp insertion in *d3-2* occurred (Fig. 5A). Different alleles present same phenotype, and *d2-1* and *d3-1* were used for further characterization. As shown in Fig. 5B, *d2* exhibited greener leaves than wild type, while the leaf colors of *d3* and wild type are comparable. Accordingly, photosynthetic pigment contents increased significantly in *d2* and slightly in *d3* (Fig. 5C). Net photosynthesis rate in *d2* is significantly higher than that of wild type and unchanged in *d3* (fig. S9). We also overexpressed *DGP2*/*3* in wild-type background to test their biological function. Measurements of gene expression level confirmed the up-regulation of *DGP2* in *DGP2* overexpression (*D2*-OE) and *DGP3* in *DGP3* overexpression (*D3*-OE; fig. S10). *D2*-OE and *D3*-OE displayed yellowish leaves from the seedling stages, which contained significant less photosynthetic pigment and presented significantly decreased net photosynthesis rate (Fig. 5D and figs. S9 and S11). The panicles of both *D2*-OE and *D3*-OE are white in color with less photosynthetic pigment (Fig. 5, E and F). These results demonstrated that *DGP2* and *DGP3* function negatively in rice chlorophyll content regulation.

On the basis of the results that DGP2/3 repress the OsGLK activity and the phenotype of *DGP2*/*3*-OE resembles that of *Osglk2*, we speculated that the expression level of OsGLK-regulated genes would be changed in *DGP2*/*3*-OE. The results of transcriptome sequencing and qRT-PCR indicated that antenna protein genes (*LHCA1*, *LHCA3*, *LHCA4*, *LHCB1*, *LHCB2*, and *LHCB4*) and chlorophyll synthesis–related genes (*HEMA1*, *CHLH*, *YGL1*, and *PORA*) are down-regulated and that *ELIP* genes (*ELIP1*-*ELIP3*) are up-regulated (Fig. 5, G to I).

### OsGLK directly activates the expression of *DGP1*/*2*/*3*

**Fig. 6.**
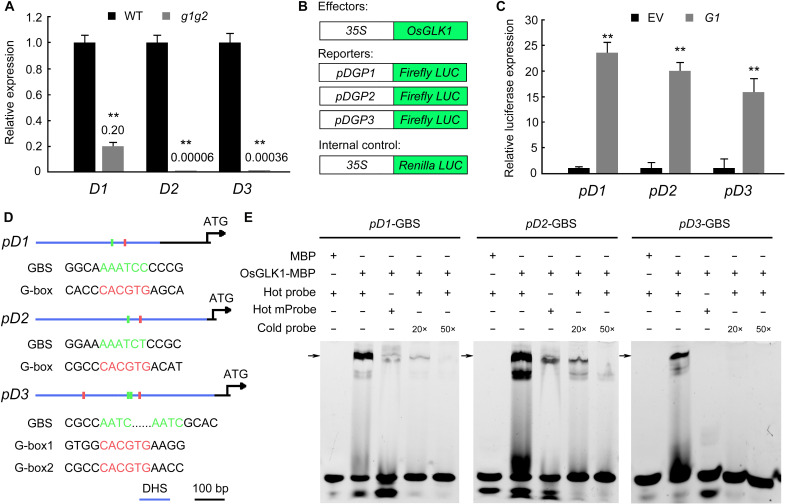
OsGLK directly activates the expression of *DGP1*/*2*/*3*. (**A**) Expression level analysis of *DGP1*/*2*/*3* in WT and *Osglk1 Osglk2*. (**B**) Schematic diagram of construct used in transactivation activity assays. Promoters are shown as white boxes, and CDSs are shown as green boxes. (**C**) Analysis of the transcription activities of OsGLK1 on promoter of *DGP1*/*2*/*3*. (**D**) Schematic diagram of promoter structure of DGP*1*/*2*/*3*. (**E**) Electrophoretic mobility-shift assays (EMSAs) showing that OsGLK1 directly binds to the GLK-binding site (GBS) of *DGP1*/*2*/*3* promoters. The hot mutated probes (mProbes) contain mutant nucleic acid from GBS to polyadenylate. The arrow heads indicate the shift bands.

When analyzing the transcriptome data of *Osglk*, we accidentally found that *DGP1*/*2*/*3* are down-regulated significantly in *Osglk1*, *Osglk2*, and *Osglk1 Osglk2* (fig. S12). In particular, the normalized read count of *DGP2* in *Osglk1 Osglk2* decreased to zero, which indicated completely gene silencing. These down-regulations of *DGP1*/*2*/*3* in *Osglk1 Osglk2* were verified by qRT-PCR assay (Fig. 6A). Dual-luciferase reporter assays proved that both OsGLK1 and OsGLK2 activate the expression of *DGP1*/*2*/*3* (Fig. 6, B and C, and fig. S13).

To test whether OsGLKs activate the expressions of *DGP1*/*2*/*3* directly, we analyzed the deoxyribonuclease hypersensitive site (DHS) of *DGP1*/*2*/*3* promoters, which are typically inferred as open chromatin and are accessible to regulatory proteins (*43*). G-box (CACTGT) and GLK-binding sites (GBSs; RAATCY, R = A/G, Y = T/C), both of which were predicted as targets of GLKs, were identified in the DHS of *DGP1*/*2* promoters (*10*, *44*). The DHS of *DGP3* also harbors G-box and two unstrict GBSs (Fig. 6D). Electrophoretic mobility-shift assays (EMSAs) showed that OsGLK1 is able to bind to the labeled probes of GBSs in *DGP1*/*2*/*3* promoters, and the shifted band signals were substantially weakened upon application of the unlabeled cold probes or hot probes with mutated nucleotide (Fig. 6E). However, no shifted band signals were observed when G-boxes were used as probes (fig. S14).

As DGP1/2/3 repress the activities of OsGLK and OsGLK directly activate the expressions of *DGP1*/*2*/*3*, overexpression of one *DGP* homolog would down-regulate other ones. To test this, we inquired our transcriptome data of glumes. As expected, the expression levels of *DGP1* and *DGP3* are significantly decreased in *D2-OE* plants. In addition, the expression levels of *DGP1* are significantly decreased in *D3-OE* plants. No transcripts of *DGP2* were detected in glumes of wild type and *D3-OE* plants (fig. S15). These results indicated that OsGLKs bind to the promoters of *DGP1*/*2*/*3* and activate their expressions.

### OsGLK-DGP module regulates rice grain size and yield

**Fig. 7.**
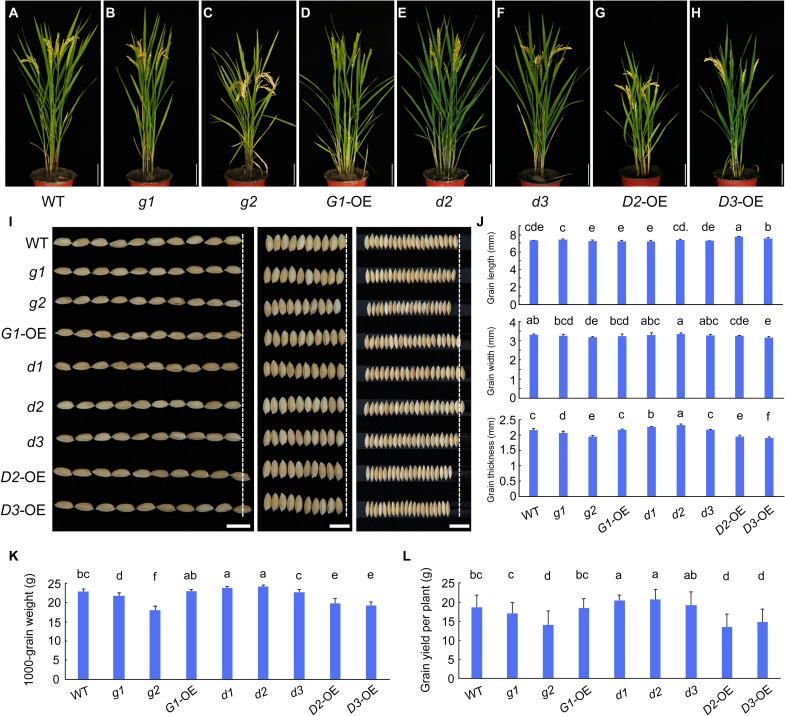
Phenotypic characteristics of overexpression lines and mutants of *OsGLK* and *DGP*. (**A** to **H**) Mature plants of overexpression lines and mutants of *OsGLK* and *DGP*. Genotypes are listed below. Scale bars, 10 cm. (**I**) Grains of overexpression lines and mutants of *OsGLK* and *DGP*. Genotypes are listed on the left. Scale bars, 1 cm. (**J**) Comparison of grain length, grain width, and grain thickness between different genotypes. (**K** and **L**) Comparison of 1000-grain weight and grain yield per plant between different genotypes. Values are shown as means ± SD (*n* = 20 plants). Different lowercase letters indicate significant differences [*P* < 0.05; one-way analysis of variance (ANOVA) with least significant difference test].

As photosynthesis is closely related to plant growth and developments, overexpression lines and mutants of *OsGLK* and *DGP*, including *Osglk1*, *Osglk2*, *OsGLK1*-OE, *dgp1*, *dgp2*, *dgp3*, *DGP2-OE*, and *DGP3-OE*, were grown under natural paddy field conditions for a phenotypic analysis. Compared with wild type, plant height decreased significantly in *Osglk2*, *DGP2-*OE, and *DGP3-*OE (Fig. 7, A to H, and fig. S16A). Panicle number, panicle length, and grain number per panicle are comparable between wild type and plants with different genotypes (fig. S16, B to D). *OsGLK1-*OE plants showed significantly decreased seed-setting rate, which is consistent with the results of a previous study (fig. S16E) (*27*).

We found that grain shape and yield are influenced by OsGLK-DGP module. Compared with wild type, the grain length of *DGP2-*OE and *DGP3-*OE increased, and the grain width of *Osglk2*, *DGP2-*OE, and *DGP3-*OE decreased. Grain thickness of *Osglk1*, *Osglk2*, *DGP2-*OE, and *DGP3-*OE decreased. Meanwhile, *dgp1* and *dgp2* displayed significantly increased grain thickness (Fig. 7, I and J). One thousand–grain weight of *Osglk1*, *Osglk2*, *DGP2-*OE, and *DGP3-*OE decreased by 4.51, 20.73, 13.06, and 16.00%, respectively. *dgp1* and *dgp2*, conversely, showed increased (4.05% for *dgp1* and 5.75% for *dgp2*) 1000-grain weight (Fig. 7K). Similarly, grain yield per plant decreased in *Osglk2*, *DGP2-*OE, and *DGP3-*OE but increased in *dgp1* and *dgp2* (Fig. 7L).

## DISCUSSION

**Fig. 8.**
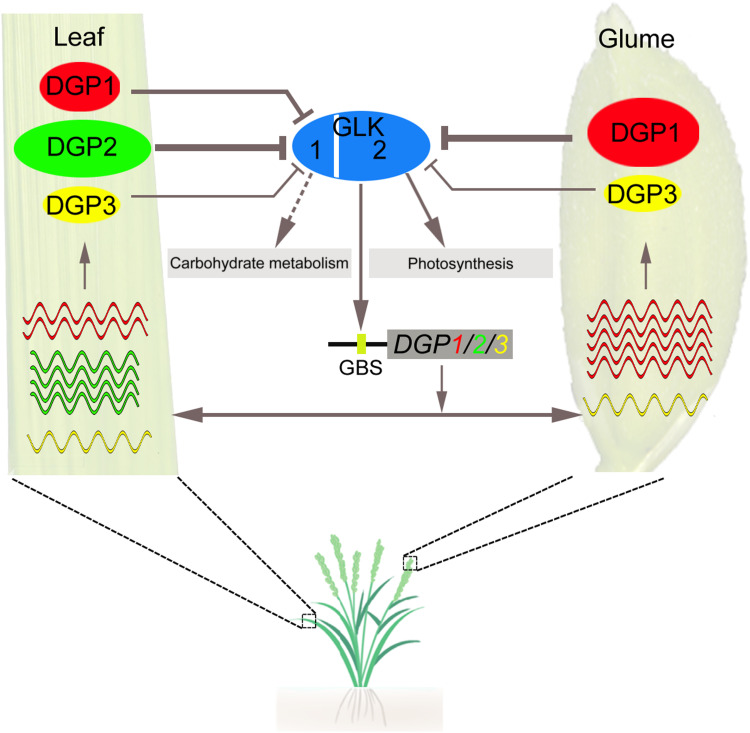
A working model of the OsGLK-DGP feedback module. OsGLK1/2 regulate the expression of photosynthesis-related genes directly and carbohydrate metabolism–related genes indirectly in a partially redundant manner, with OsGLK2 playing a more dominant role. OsGLK binds the GBS of *DGP1*/*2*/*3* promoters to activate their expression. DGP1/2/3 interact with OsGLK1/2 to repress their activities. *DGP1*, *DGP2*, and *DGP3* displayed differentiated expression pattern in leaf and glume. In leaves, *DGP2* is the dominantly expressed gene within these three genes, and the expression level of *DGP1* and *DGP3* is relatively low. Mutation of *DGP2* resulted in a greener leaf phenotype. In glumes, *DGP1* is highly expressed, and *DGP3* is expressed in a low level. *DGP2* is not expressed in glumes. Mutation of *DGP1* leads to dark-green panicles.

Increasing photosynthetic efficiency is an important means to boost crop yield. Plant GLKs are central regulators of photosynthesis, which makes the GLKs promising targets for crop breeding. In this study, we reported a feedback module in which DGP1/2/3 interact with OsGLK1/2 to repress their transcriptional activity. Meanwhile, OsGLK1/2 directly bind to the promoters of *DGP1*/*2*/*3* to activate their expressions (Fig. 8).

### Partially redundant role of OsGLK in rice photosynthesis regulation

It has been documented that GLK pairs play redundant role in photosynthesis regulation, especially in C_3_ plants. In *Arabidopsis*, *Atglk1* and *Atglk2* are essentially indistinguishable from wild type throughout most of development stages (*8*). However, the researcher noted that *Atglk2* mutant plants exhibit pale green siliques, which may due to expression repression of *AtGLK1* in this organ. In our study, we found that disruptive mutation of both *OsGLK1* and *OsGLK2* leads to chlorosis phenotype, which is more prominent in panicles than in leaves (Fig. 1, A to F). Moreover, the *Osglk1 Osglk2* double mutants present a severer phenotype and are seedling lethal. TEM observation showed chloroplast development defects in both *Osglk2* and *Osglk1 Osglk2*, in accordance with their macroscopic phenotypes (Fig. 1, G to J). The partial redundancy of OsGLK1 and OsGLK2 was also validated by transcriptome results. The reduction extent of chlorophyll synthesis–related genes is marked in *Osglk1 Osglk2*, moderate in *Osglk2*, and slight in *Osglk1* (Fig. 2E). Carbohydrate metabolism genes were enriched in DEGs (Fig. 2, D to F). It is reasonable, as photosynthesis participates in this process. However, this regulation may be indirect, as these carbohydrate metabolism genes are barely identified as targets of OsGLK (*11*). In a previous functional study on *OsGLK*, RNAi-induced *Osglk1* and T-DNA insertion–triggered *Osglk2* are phenotypically same to wild type and *Osglk1 Osglk2* is viable (*13*). We speculate that these differences with our results are caused by residual protein activity, as both RNAi and T-DNA insertion may not be fully penetrant.

Overexpression of *OsGLK1* and *OsGLK2* leads to panicle-specific darker green phenotype, mimicking the phenotype of *dgp1* (Fig. 3 and fig. S3) (*41*). As the transcriptional level is indeed increased in leaves, we speculate that protein activities of OsGLK in overexpression plant leaves are repressed, probably by DGP2, which exhibit leaf-exclusive expression manner (see below).

### OsGLK and DGP1/2/3 form a feedback regulation module

In previous studies, we identified DGP1 as a repressor of OsGLK activity and photosynthesis (*41*). Here, we proved that DGP2/3, two factors with similar sequence and protein length with DGP1, exert effect on rice photosynthesis in a same manner. Both DGP2 and DGP3 interact with the GCT domain of OsGLK (Fig. 4, A and B). BiFC results showed that DGP2/3 interact with OsGLK1/2 throughout the cells. Also, DGP2 and DGP3 displayed a ubiquitous distribution pattern in rice protoplast (fig. S6). Whether the interactions between OsGLK and DGP2/3 functions beyond regulation of nuclear gene expression is currently unknown. Transactivation assays showed that DGP2/3 repress the activation activity of OsGLK2 on its target genes (Fig. 4, C to E). Knockout of *DGP2* and *DGP3* leads to greener leaves (Fig. 5, A to C). Conversely, overexpression of *DGP2* and *DGP3* produces faded leaves and white panicles, in accompany with down-regulation of antenna protein and chlorophyll synthesis genes (Fig. 5, D to I).

Beside DGP2 and DGP3, four other DGP1 homologs, named DGP4 to DGP47 were identified (table S1). All these four proteins harbor the TIGR01589 domain but longer than DGP1/2/3 in length (fig. S5). Homolog searching indicated that DGP4/7 show similarity with *Arabidopsis* histidine-tRNA ligase. DGP5/6 show homologies with zinc finger–containing helicase protein. Whether these proteins participate in photosynthesis regulations or other biological process is currently obscure and warrant more studies.

Although DGP1/2/3 function in similar molecular pathways, the phenotypes of *dgp1*, *dgp2*, and *dgp3* are divergent. As the gene name *DEEP GREEN PANICLE* indicated, the phenotype of *dgp1* was exclusively observed in panicles (*41*). In contrast, *dgp2* displays greener leaves without obvious color change in panicles. As for *dgp3*, minor color change is observed in leaves and not in panicles. Querying our transcriptomics data of rice leaves and panicles, we found that *DGP1* is predominantly expressed in panicles [Fragments Per Kilobase of exon model per Million mapped fragments (FPKM) value is 62 in leaves and 384 in panicles], and *DGP2* is exclusively expressed in leaves (FPKM value is 102 in leaves and 0 in panicles). DGP3 displays relatively low expression level in both leaves and panicles (FPKM value is 33 in leaves and 3.3 in panicles) (fig. S17). We thus speculate that phenotype differences between *dgp1*, *dgp2*, and *dgp3* are mainly caused by differentiated expression patterns of these three genes (Fig. 8).

Feedback regulation is a common and efficient regulatory mechanism in organisms. Our results demonstrated that OsGLKs bind to the promoters of *DGP1*/*2*/*3* and activate their expression (Fig. 6). These direct activations and the repression effect of DGP1/2/3 on OsGLK form a feedback regulation module (Fig. 8). In this module, the activities of OsGLK are fine-tuned within a ‌homeostasis‌. When external signals (for example, light) or internal factors increase the expression level of *OsGLK* or the activity of OsGLK, elevated OsGLK activity will enhance the transcription of *DGP1*/*2*/*3*. More DGP1/2/3 accumulation then interacts with OsGLK to reduce their activities. Oppositely, when the expression level of *OsGLK* or activities of OsGLK decreased, accumulation of DGP1/2/3 will reduce, and activities of OsGLK are released.

### Mutation of *DGP1*/*2* increases rice yield

Manipulation of *GLK* expression has been implicated in boosting crop yields. Constitutive expression of maize *GLK* genes in rice resulted in increased carbohydrate levels and a 30 to 40% increase in both vegetative biomass and grain yield (*20*). Rice transformed with maize *ZmGLK1* and *ZmGLK2* showed increases in grain yield markedly. However, promoter of *Ubiquitin*-drived *ZmGLK1* produced smaller seeds without yield increases (*21*). Endosperm-specific overexpression of rice *OsGLK1* leads to increased grain yield but deteriorated grain quality in rice (*26*). Heterologous expression of sunflower *HaGLK* enhances photosynthetic efficiency, crop yields, and stress resilience in rice (*22*). Overexpression of *OsGLK1* in rice resulted in low seed-setting rates (fig. S16E) (*27*). This hinders the breeding utilization of *OsGLK* in a transcriptional level.

Our results showed that mutations of *DGP1* and *DGP2* increase seed thickness, 1000-grain weight, and grain yield per plant without fertility cost. On the basis of our results, DGP1 and DGP2 are dominant repressors of OsGLK activities in glumes and leaves, respectively. Both glume and leaf are source organs that synthesize carbohydrates by photosynthesis. We speculate that mutation of *DGP1* and *DGP2* increased the activities of OsGLK and enhanced the photosynthetic capacity. More carbohydrates were produced and translocated to seeds. In support of this, net photosynthesis rate is increased in *dgp2* leaves, and the contents of sucrose, the product of photosynthesis and the main form of transport carbohydrate within plants, increased in leaves of *dgp2* and glumes of *dgp1* (figs. S9 and S18). Then, size and weight of seeds increased accordingly. We hypothesize that combination of *DGP1* and *DGP2* mutations would increase rice yield further, as they are expressed dominantly in different organs. Moreover, this “repressor removal” strategy for improving photosynthesis and yield may also be effective in other crops.

## MATERIALS AND METHODS

### Plant materials

The mutants of *Osglk1 Osglk2*, *dgp2*, and *dgp3* were generated by targeted mutagenesis in the japonica rice (*Oryza sativa*) variety Yandao 8 using CRISPR-Cas9 technology (*45*). The corresponding single mutants were segregated from the double heterozygous mutant. The *dgp1* used in this study was reported previously (*41*). For overexpression assay, coding sequence (CDS) of genes (*OsGLK1*, *OsGLK2*, *DGP2*, and *DGP3*) was inserted into pCAMBIA23A vector with rice actin promoter. The resulting constructs were sequenced and transformed into *Agrobacterium tumefaciens* EHA105 and then into calli of Yandao 8. The variety Yandao 8 was used as the wild type in all experiments. All plant materials were grown in paddy fields in Yangzhou (Jiangsu Province, China) or Lingshui (Hainan Province, China).

### Measurement of photosynthetic pigment content

Photosynthetic pigment contents were determined by the previous method with minor modifications (*41*). A total of 0.1 g of fresh leaves was cut and soaked in a 10-ml solution of 1:1 ethanol/acetone (v/v). After 12 hours of treatment in darkness, the absorbance values at 470-, 645-, and 663-nm wavelength were measured using a microplate reader. The pigment contents were calculated on the basis of the absorbance values as follows: Chl a (Chlorophyll a) = (12.72 × A_663_ – 2.69 × A_645_) × 10/(1000 × 0.1); Chl b = (22.9 × A_645_ – 4.68 × A_663_) × 10/(1000 × 0.1); Car (Carotenoid) = [4.7 × A_470_ – (0.27 × Chla + 0.27 × Chlb)] × 10/(1000 × 0.1). All experiments were carried out with three biological replicates.

### TEM analysis

Fresh leaves of rice were cut into pieces and fixed in 3% glutaraldehyde in phosphate buffer. After washing three times with phosphate buffer (0.1 M), the samples were fixed in 1% OsO_4_ for 1.5 hours. After fixation, the samples were washed again three times in phosphate buffer (0.1 M). The samples were dehydrated through an acetone series (50% for 15 min, 70% for 15 min, 90% for 15 min, and 100% for 20, 20, and 20 min) and ultimately embedded in Epon 812 resin (acetone: embedding fluid = 2:1 for 0.5 hours, acetone: embedding fluid = 1:2 for 1.5 hours at 37°C, embedding fluid for 3 hours at 37°C). After solidifying at 37°, 45°, and 60°C for 24 hours, ultrathin sections (70 nm) were produced using an LKB-V slicer. The samples were double stained with uranyl acetate and lead citrate for 15 min, respectively. The sections were then observed with a JEOL-1200E transmission electron microscope and documented by Morada G2.

### Measurement net photosynthetic rate

Net photosynthetic rate was measured in flag leaves of field-grown rice at the heading stage with a LI-6800 instrument (LI-COR Biosciences, USA). The CO_2_ concentration was set to 400 μmol/mol, and the photosynthetic photon flux density was set to 1200 μmol/m^2^ per second. Six plants were randomly selected for measurement.

### Measurement of sucrose content

Flag leaves and glumes of field-grown rice at the heading stage were used to measure the sucrose content. Samples were weighed and thoroughly ground with liquid nitrogen‌. Sucrose content was determined using a plant sucrose content detection kit (BC2460, Solarbio, Beijing) and visible spectrophotometry. Six plants were randomly selected for measurement.

### Transcriptome sequencing analysis

Transcriptome sequencing and partial data analysis was performed by Shanghai Personal Biotechnology Cp. Ltd. Briefly, total RNA was isolated using the TRIzol Reagent (Invitrogen Life Technologies). Three micrograms of RNA was used as input material for the RNA sample preparations. Sequencing libraries were generated according to the following steps. First, mRNA was purified from total RNA using oligo(dT)-attached magnetic beads. Fragmentation was carried out, and first strand cDNA was synthesized using random oligonucleotides and SuperScript II. Second strand cDNA synthesis was subsequently performed using DNA polymerase I and ribonuclease H. DNA fragments with ligated adaptor molecules on both ends were selectively enriched using Illumina PCR Primer Cocktail in a 15-cycle PCR reaction. The sequencing library was then sequenced on NovaSeq 6000 platform (Illumina).

### qRT-PCR assay

Total RNA was extracted from rice tissues using TRIzol reagent. Reverse transcription was performed using HiScript III RT SuperMix for RT-qPCR (+gDNA wiper, Vazyme). Real-time PCR analysis was performed using ChamQ SYBR qPCR Master Mix (Vazyme) and the Bio-Rad CFX96 real-time PCR instrument. All reactions were performed in triplicate, with *Ubiquitin* as the normalized reference gene for all comparisons.

### Subcellular localization

The CDS of *DGP2*/*3* was amplified and cloned into pJIT163-GFP vector (Biovector, Beijing, China) in frame with green fluorescent protein (GFP). The OsbZIP52-RFP fusion protein was used as a nuclear localization marker in the assay. Plasmids were transfected into rice protoplasts through polyethylene glycol (PEG)–mediated transformation. Empty pJIT163-GFP vector was used as the control. Fluorescence signals were captured at 24 hours after transformation with a confocal laser scanning microscope (Carl Zeiss LSM 710, Oberkochen, Germany). GFP and red fluorescence signals were excited at 488 and 561 nm, and emissions were collected at 500 to 540 and 600 to 650 nm, respectively.

### Yeast two-hybrid assay

The CDSs of DGP2/3 and OsGLK1/2 were amplified and cloned into the pGADT7 and pGBKT7 vectors. The transformations were conducted using Frozen-EZ Yeast Transformation II kit according to the manufacturer’s instructions. Double dropout medium (SD/−Leu-Trp) was used to verify the cotransformation, and quadruple dropout medium (SD/−Leu-Trp-His-Ade) was used to test the interactions. The yeast strain used in yeast two-hybrid assays was Y2HGold.

### BiFC assay

The CDSs of *DGP2/3* and *OsGLK1/2* were amplified and cloned into the Venus N and Venus C vectors, respectively. The plasmid pairs were cotransfected into protoplasts of young rice seedling by 40% (w/v) PEG. Transfected protoplasts were incubated in the dark at 28°C overnight and observed using a laser scanning confocal microscopy (Nikon AX). Yellow fluorescent proteins (YFPs) were excited at 511 nm, and emissions were collected at 525 nm.

### Transactivation activity assays

For the effect of DGP2/3 on OsGLK2 activity, the 2-kb DNA fragments of the promoter regions of *OsLHCB4* and *OsHEMA1* were amplified and cloned into the pGreen II 0800-LUC vector, respectively. The CDS of *OsGLK2* was fused into the pGreen II 62-SK vector (*46*). The construct for subcellular localization, pJIT163-DGP2/3-GFP, was used to induce the expression of *DGP2* and *DGP3*. For the effect of OsGLK1/2 on *DGP1*/*2*/*3* activation, the 2-kb DNA fragments of the promoter regions of *DGP1*/*2*/*3* were amplified and cloned into the pGreen II 0800-LUC vector, respectively. The construct pGreen II 62-SK fused with CDS of *OsGLK1*/*2* was used to induce the accumulation of OsGLK1/2.

The different combinations of vectors were cotransfected into rice protoplasts by PEG-mediated transformation. After incubation in the dark at 28°C for 20 hours, protoplasts were harvested and lysed for the detection of firefly luciferase and renilla luciferase activities. The ratios of firefly luciferase to renilla luciferase activity were calculated to define relative promoter activity. Data were obtained from three replicates.

### Electrophoretic mobility-shift assay

The CDS of *OsGLK1* was amplified and inserted into pMAL-c5X vector to generate OsGLK1–MBP (maltose-binding protein) construct. On the basis of the sequence of the *DGP1*/*2*/*3* promoter, unique 33-bp single-stranded oligo containing the GBS or G-box was synthesized and labeled with FAM (fluorescein amidite) at 5′ end (Sangon Biotech, Shanghai, China). The same sequence without FAM label served as the competition probe. Mutated probes (mProbe) were synthesized by replacing the GBS or G-box with poly adenine. Purified OsGLK1-MBP protein was incubated with the probe for 30 min. The DNA-protein complex was separated by a 6% native polyacrylamide gel electrophoresis gel using 0.5 × TBE (Tris-borate-EDTA) buffer. After 1 hour of electrophoresis, the gel image was captured using a FluorChem M system (ProteinSimple, San Jose, USA).
