# Supplementary material for: An OsGLK1/2-DGP1/2/3 feedback module regulates rice photosynthesis and yield
Source: Sci Adv. 2026 Jun 24;12(26):eaef5234. doi: 10.1126/sciadv.aef5234 (PMC13292954; doi:10.1126/sciadv.aef5234)
Supplement: Supplementary file 1 — Figs. S1 to S18 Table S1 [file sciadv.aef5234_sm.pdf]

Science Advances
AAAS

Supplementary Materials for

An OsGLK1/2-DGP1/2/3 feedback module regulates rice photosynthesis and yield

Xueju Liu *et al.*

Corresponding author: Hengxiu Yu, hxyu@yzu.edu.cn

Sci. Adv. **12**, eaef5234 (2026)
DOI: 10.1126/sciadv.aef5234

This PDF file includes:

Figs. S1 to S18
Table S1

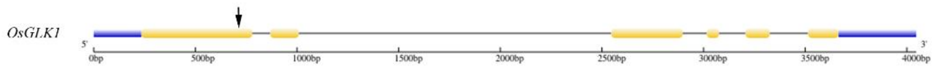

WT GCCGTCATCGACGACGTCGTCGTCGCAGGAGGCTGAGAGCCGGCACAAGTCATCCAGCAAGAGCTCCCATGGGAAGA

Osglk1-1 GCCGTCAT- -GGGAAGA

|← 62 bp →|

Osglk1-2 GCCGTCATCGACGACGTCGTCGTCGCAGGAGGCTGAGAGCCGGCACAAGTCATCCAGCAAGAGCTCACCATGGGAAGA

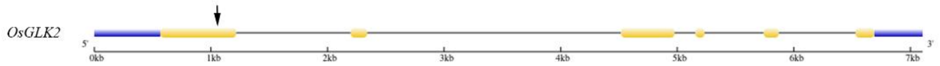

WT AACGGCGGCGGCGACGAGGTCCTGAGCGCGGTGACGACGGAGGATTCCTCGG

Osglk2-1 AACGGCGGCGGCGACGAGGTCCTGA - - - - - - - - - - - - - - - - - - -TTCCTCGG

|← 19 bp →|

Osglk2-2 AACGGCGGCGGCGACGAGGTCCTGAAGCGCGGTGACGACGGAGGATTCCTCGG

Fig. S1 Gene structures of *OsGLKs* and genotypes of *Osglk* mutants

Coding regions are shown as yellow boxes. 5’- and 3’-untranslated regions are shown as blue boxes. Introns are shown as black lines. The arrow indicates the position of target sequence for CRISPR-Cas9. Details of sequence modification in each mutant are listed below.

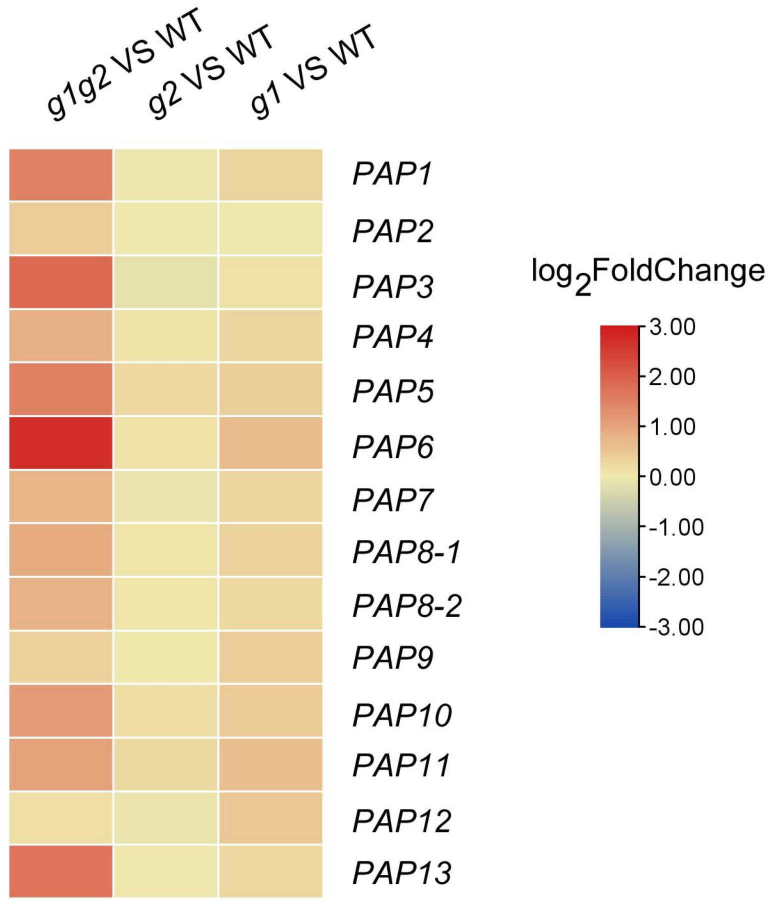

Fig. S2 Heatmap of the fold changes ($\log_2$) in the expression of *PAPs* genes in each comparison

Data of normalized read counts from transcriptome analysis were used to calculate the expression levels.

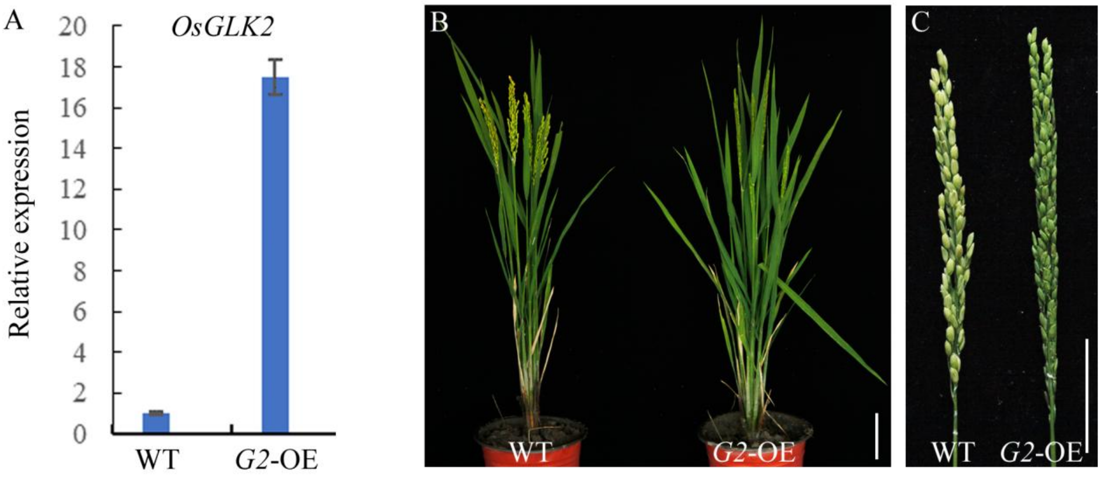

Fig. S3 Characterization of *OsGLK2* over expression plants

(A) Relative expression levels of *OsGLK2* in wild-type and *G2*-OE (*OsGLK2* over expression). (B) Comparison of the plants of wild type (WT) and *G2*-OE. Scale bar=10 cm. (C) Comparison of the panicles of wild type (WT) and *G2*-OE. Scale bar=5 cm.

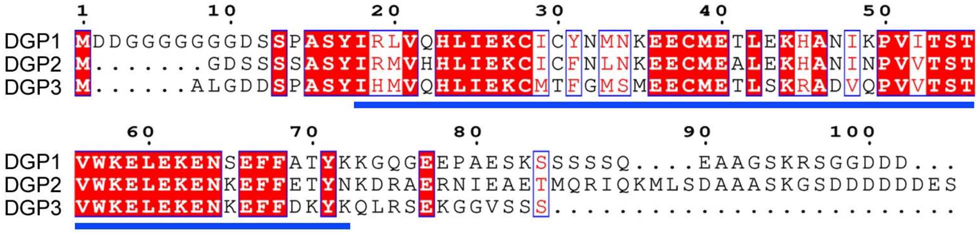

Fig. S4 Multiple sequence alignment of DGP1, DGP2, and DGP3

Identical amino acids are shaded in red. The TIGR01589 domain are underlined in blue

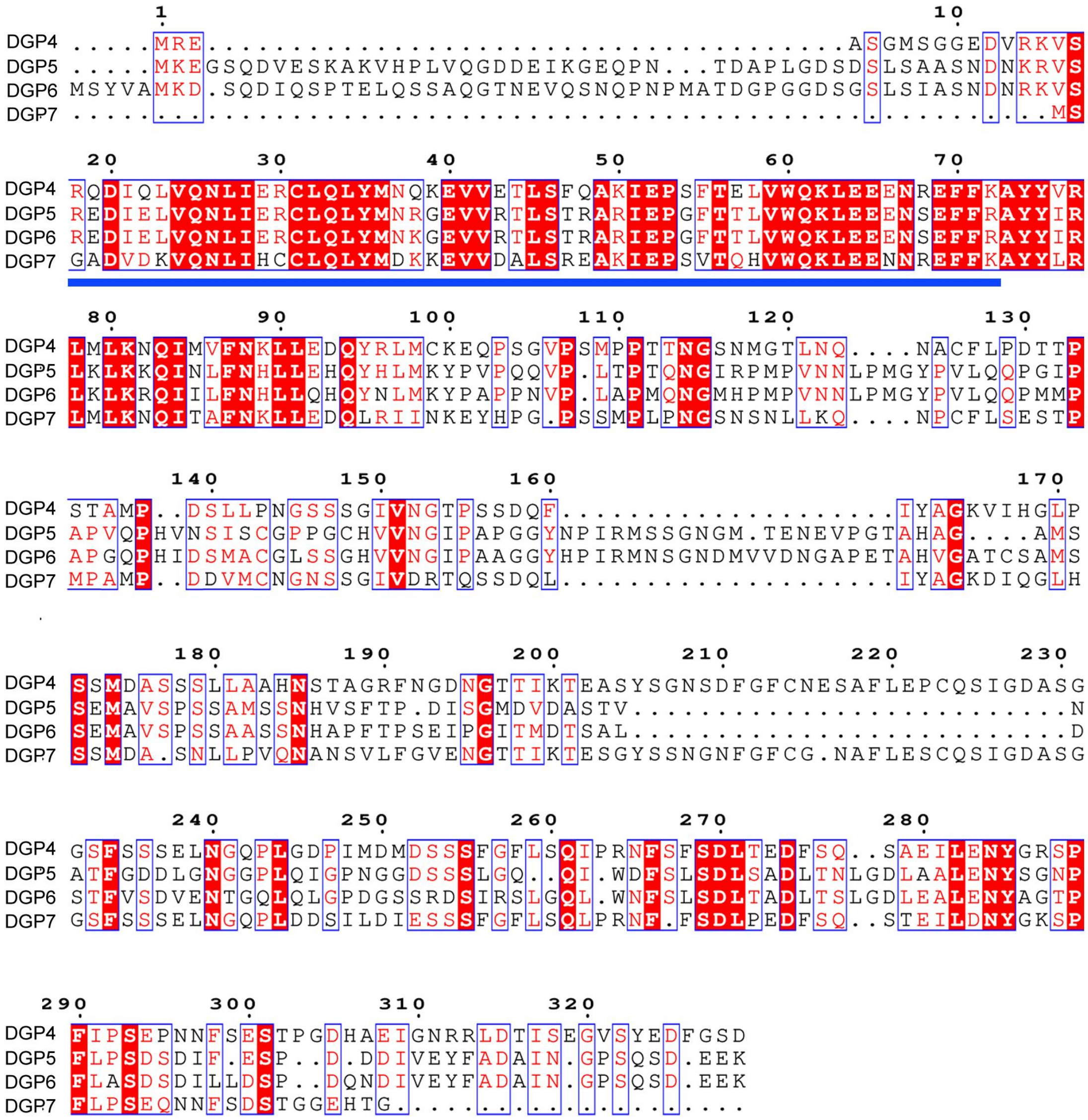

Fig. S5 Multiple sequence alignment of DGP4, DGP5, DGP6, and DGP7

Identical amino acids are shaded in red. The TIGR01589 domain are underlined in blue

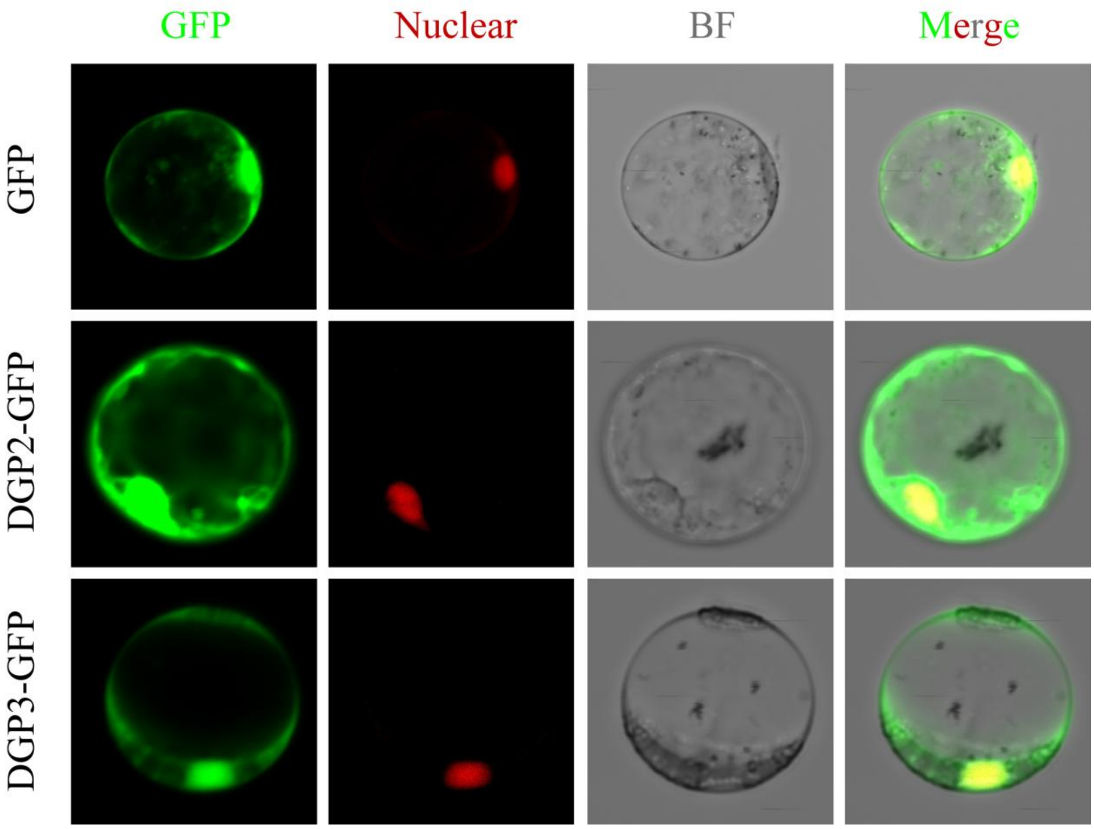

Fig. S6 Subcellular localization of DGP2 and DGP3 in rice protoplasts

The fused protein OsbZIP52-RFP (red fluorescent protein) is used as the nuclear marker. BF, bright field.

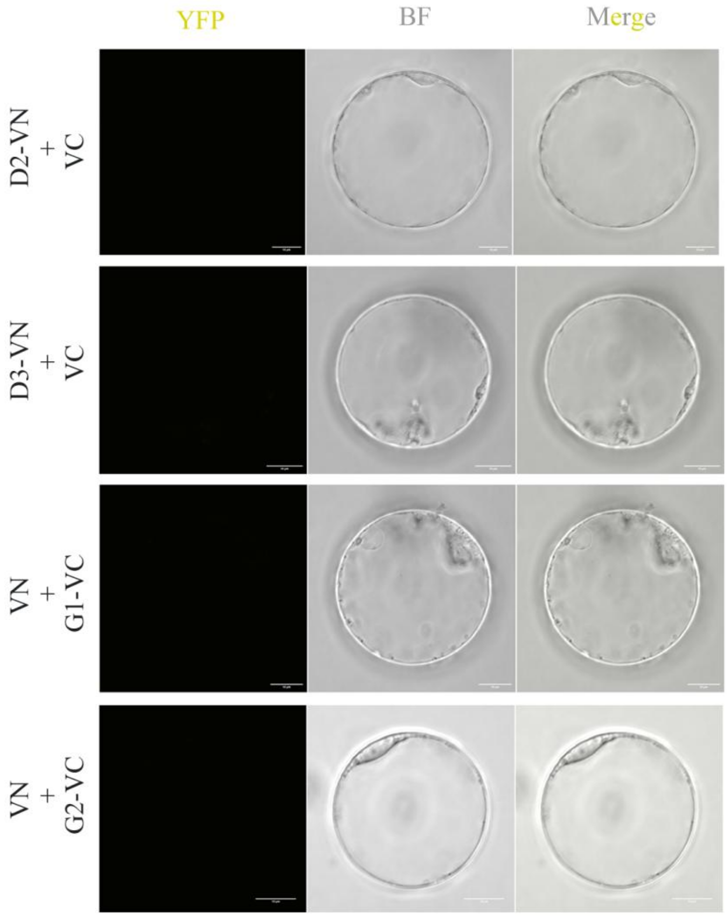

Fig. S7 No interactions between DGP2/3, OsGLK1/2 and corresponding empty vectors are observed in BiFC assay

YFP, yellow fluorescent protein. BF, bright field.

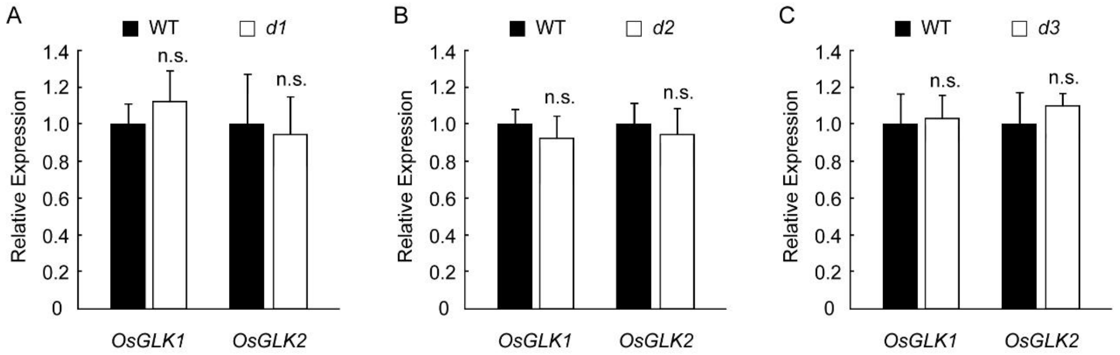

Fig. S8 Expression level analysis of *OsGLK1/2* in *dgp* mutants

(A) Expression level analysis of *OsGLK1/2* in wild type and *dgp1* glumes. (B) Expression level analysis of *OsGLK1/2* in wild type and *dgp2* flag leaves. (C) Expression level analysis of *OsGLK1/2* in wild type and *dgp3* flag leaves. n.s., no significance according to a Student's *t*-test.

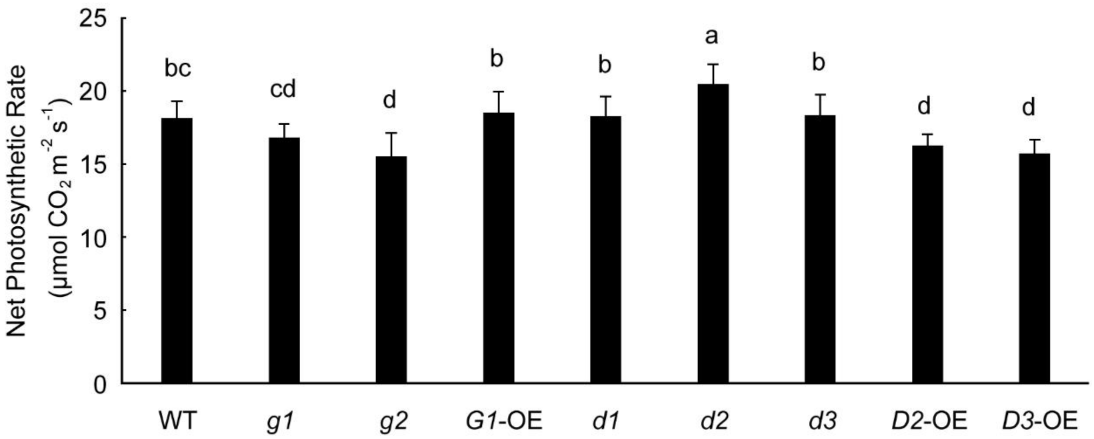

Fig. S9 Comparison of net photosynthesis rate of leaves in different genotypes

Different lowercase letters indicate significant differences ($P < 0.05$, one-way ANOVA with LSD test).

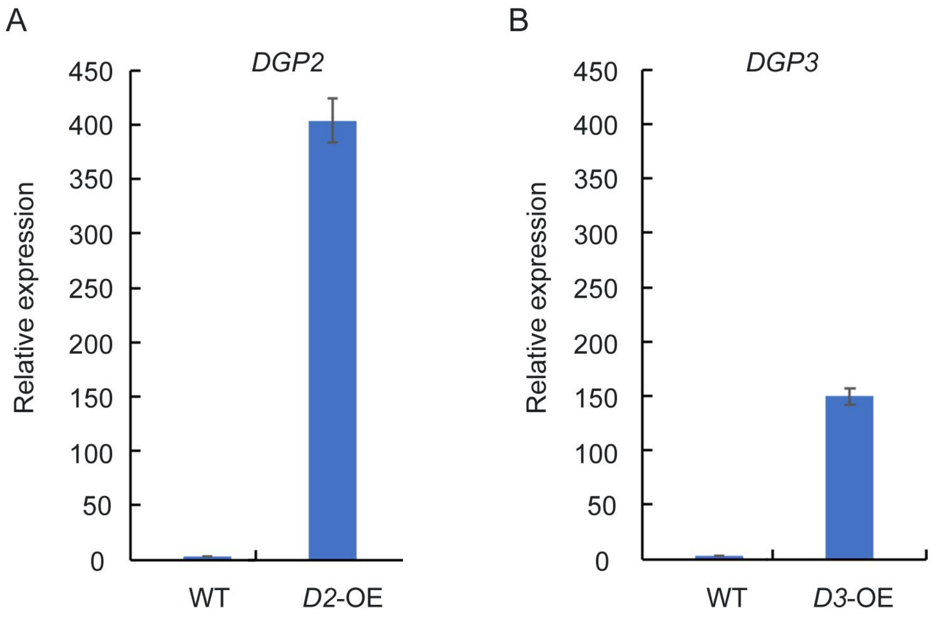

Fig. S10 Quantification of *DGP2/3* expression levels in over expression lines

(A) Relative expression levels of *DGP2* in wild type and *D2*-OE (*DGP2* over expression). (B) Relative expression levels of *DGP3* in wild type and *D3*-OE (*DGP3* over expression).

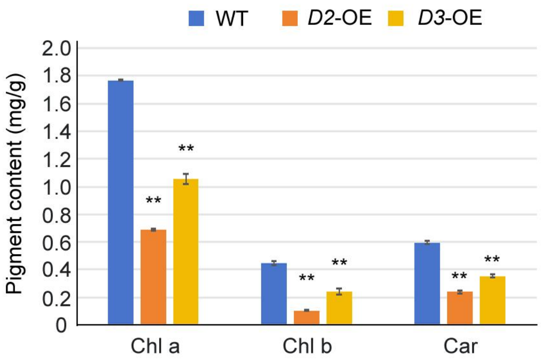

Fig. S11 Chlorophyll content in seedlings of wild type (WT), *D2*-OE, and *D3*-OE

Mean and SD values were obtained from three biological replicates. Asterisks indicate significant differences according to a Student's *t*-test (***P*<0.01).

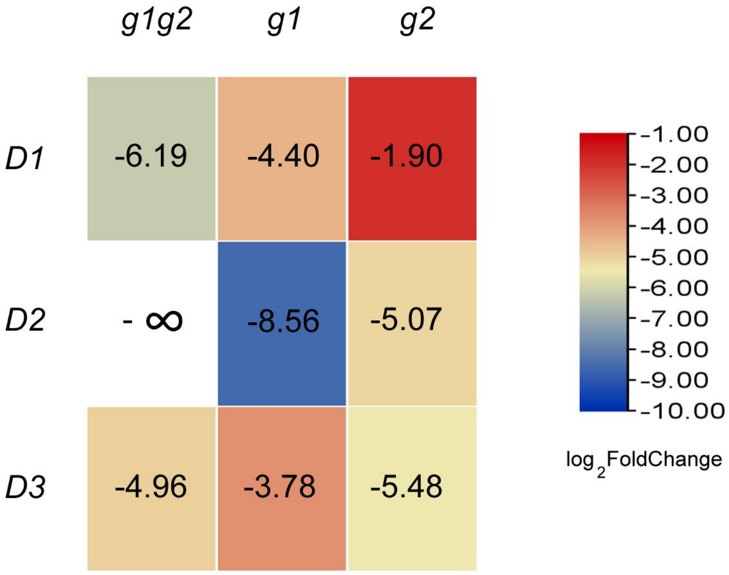

Fig. S12 Heatmap of the fold changes (log_2) in the expression of *D1/D2/D3* genes in each mutant

Data of normalized read counts from transcriptome analysis were used to calculate the expression levels. -∞, negative infinity.

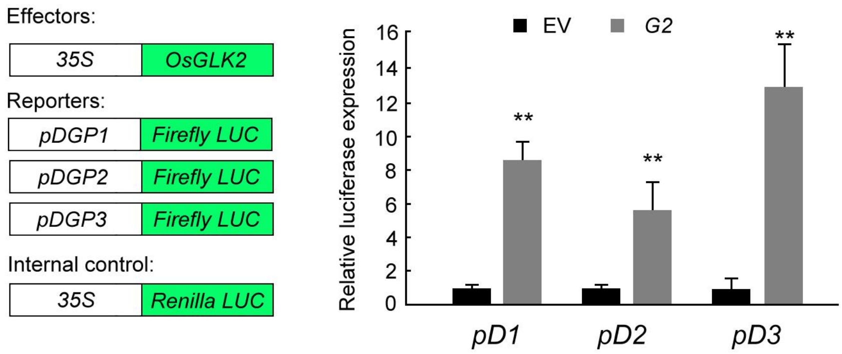

Fig. S13 OsGLK2 activate the expression of *DGP1*/*2*/*3*

Schematic diagram of construct used in transactivation activity assays are shown. Promoters are shown as white boxes and CDS are shown as green boxes. Asterisks indicate significant differences according to a Student's *t* test (***P* <0.01). EV, empty vector

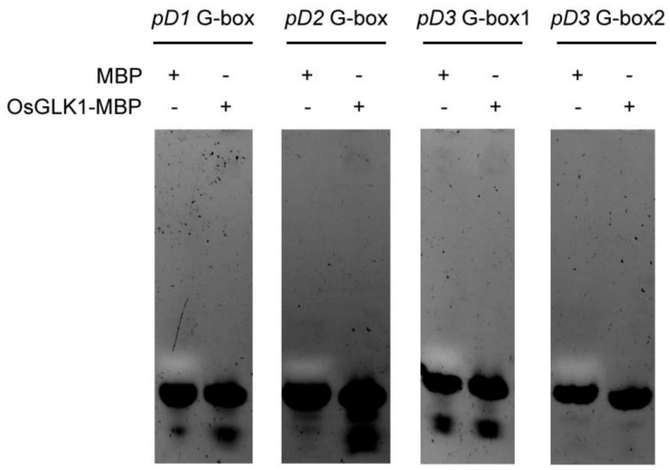

Fig. S14 OsGLK1 do not bind to G-box of *DGP1*/*2*/*3* promoters in EMSA

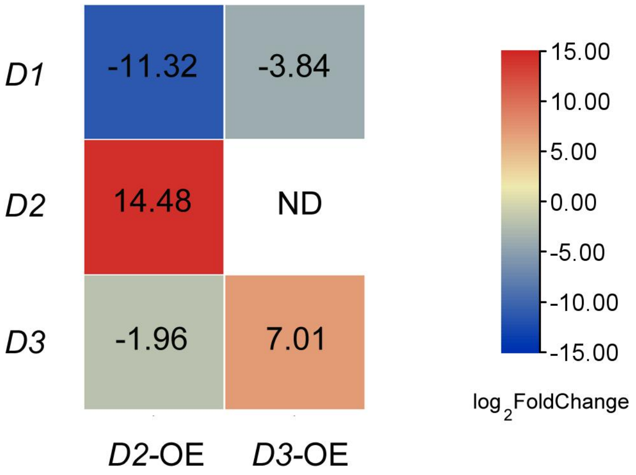

Fig. S15 Heatmap of the fold changes (log_2) in the expression of *D1/D2/D3* genes in *D2*-OE and *D3*-OE

Data of normalized read counts from transcriptome analysis were used to calculate the expression levels. ND, not detected.

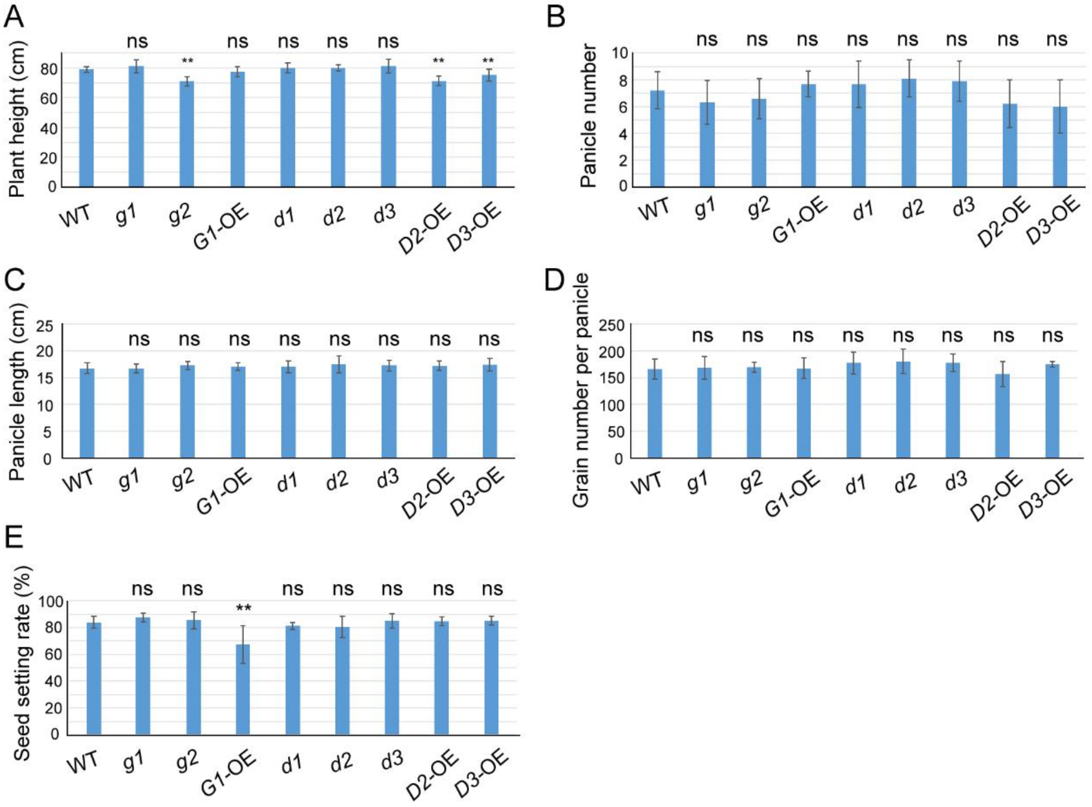

Fig. S16 Comparison of agronomic traits of rice with different genotype

(A) Plant height. (B) Panicle number. (C) Panicle length. (D) Grain number per panicle. (E) Seed setting rate. ns, no significant difference. Asterisks indicate significant differences according to a two-tailed Student's *t* test (***P*<0.01).

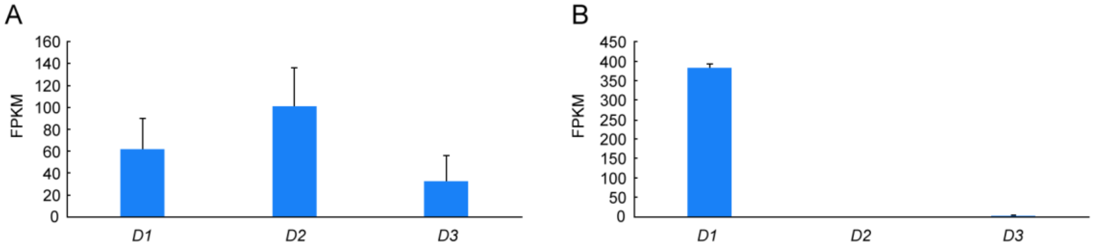

Fig. S17 Expression levels of *DGP1*/*2*/*3* in leaves and panicles

(A) Leaves. (B) Panicles. Data of FPKM (Fragments Per Kilobase of exon model per Million mapped fragments) from transcriptome analysis were used to calculate the expression levels.

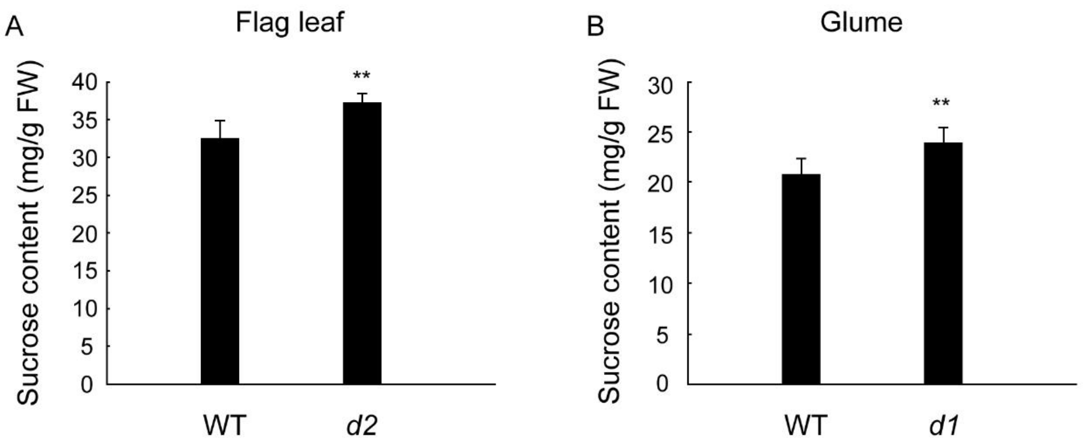

Fig. S18 Comparison of sucrose content between wild type and *dgp1/2*

(A) Comparison of sucrose content in leaves between wild type and *dgp2*. (B) Comparison of sucrose content in glumes between wild type and *dgp1*. Asterisks indicate significant differences according to a two-tailed Student's *t* test (***P*<0.01).

Table S1 Information of genes with homology to *DGP1* in rice				
Gene name	Locus identifier	Protein sequence ID	Protein size	Identities with DGP1
DGP2	LOC_Os05g38680	XP_015638792.1	102	65% (59/91)
DGP3	LOC_Os03g17200	XP_015629400.1	78	67% (46/69)
DGP4	LOC_Os02g04450	XP_015625361.1	329	52% (28/54)
DGP5	LOC_Os05g12170	XP_015637500.1	350	52% (28/54)
DGP6	LOC_Os01g12080	XP_066162940.1	314	47% (37/78)
DGP7	Os06g0705350	XP_025882092.1	289	43% (22/51)
